# Speckle tracking echocardiography in the diagnosis of early left ventricular systolic dysfunction in type II diabetic mice

**DOI:** 10.1186/1471-2261-14-141

**Published:** 2014-10-08

**Authors:** Rong-juan Li, Jiao Yang, Ya Yang, Ning Ma, Bo Jiang, Qi-wei Sun, Yi-jia Li

**Affiliations:** Department of Echocardiography, Beijing Anzhen Hospital, Capital Medical University, Beijing, China; Beijing Institute of Heart, Lung and Blood Vessel Disease, Beijing, China

**Keywords:** Diabetic cardiomyopathy, db/db mice, Echocardiography, Strain, Cardiac function

## Abstract

**Background:**

The leptin receptor-deficient db/db mouse is a well-established type II diabetes animal model used to investigate diabetic cardiomyopathy. Previous reports have documented diabetic cardiomyopathy is accompanied by cardiac structural and functional abnormalities. To better elucidate early or subtle changes in cardiac performance in db/db mice, we used speckle tracking echocardiography to assess systolic myocardial strain in vivo with diabetic db/db mice in order to study early changes of left ventricle contractile function in type II diabetes model.

**Methods:**

Male diabetic db/db mice and age-matched control mice from C57BL/6J strain at 8,12 and 16 weeks of age were subjected to echocardiography. At the midpapillary level in the parasternal left ventricular short-axis view, end diastolic and systolic left ventricular diameter, interventricular septal thickness and posterior wall thicknesses, ejection fraction, fractional shortening were determined by M-mode echocardiography. Using speckle-tracking based strain analysis of two-dimensional echocardiographic images acquired from the parasternal short-axis views at the mid-papillary level, systolic global radial and circumferential strain values were analyzed.

**Results:**

There was no significant difference in interventricular septal thickness, posterior wall thicknesses, end diastolic and systolic left ventricular diameter, ejection fraction and fractional shortening between db/db and age-matched control mice at 8,12 or 16 weeks of age (P > 0.05). At 8 and 12 weeks of age, there was no significant difference in left ventricular radial strain and circumferential strain between db/db mice and age-matched controls (P > 0.05). But at 16 weeks of age, the left ventricular radial strain and circumferential strain in db/db mice were lower than in control mice (P < 0.01).

**Conclusion:**

The present study shows that speckle tracking echocardiography can be used to evaluate cardiac functional alterations in mouse models of cardiovascular disease. Radial and circumferential strain are more sensitive and can be used for detection of early left ventricular contractile dysfunction in db/db type II diabetic mice.

## Background

The most prevalent form (90%) of diabetes mellitus is Type II (non-insulin-dependent) diabetes [1]. Epidemiological and clinical evidence shows that type II diabetes mellitus is a prevalent disease that results in a marked increase in cardiovascular complications that are in part due to a specific cardiomyopathy, characterized by ventricular dysfunction in the absence of atherosclerotic coronary heart disease or hypertension [2, 3]. Abnormalities in cardiac function and structure in diabetic subjects have been demonstrated in animal [4–7] and human studies [8–11]. However, there is still controversy regarding the severity or time of appearance of cardiac impairment following the appearance of type II diabetes.

Most reports of diabetes-induced cardiac dysfunction have used insulin-deficient (TypeI) models [12–14]. Relatively few studies on cardiac function have been conducted with type II diabetic animal models [15]. The leptin receptor-deficient db/db mouse is a well established model of type II diabetes, with obesity and insulin resistance [16, 17]. In vivo evidence for left ventricle structure and dysfunction in diabetic mice is commonly obtained from conventional echocardiography (M-mode, 2D, Doppler) [18, 19], which is typically considered late manifestations of disease and insensitive to detect subtle left ventricle dysfunction. The presence of resting abnormalities of sensitive indices of myocardial function measured by speckle tracking echocardiography (STE) has been attributed to a subclinical diabetic cardiomyopathy. Tracking the movement of myocardial tissue, STE is used to determine longitudinal strain, radial and circumferential strain for detecting global and regional myocardial dysfunction [20]. Therefore, the objective of this study was to use STE to assess systolic myocardial strain in vivo with diabetic db/db and control mice in order to study early changes of left ventricle contractile function in type II diabetes model.

## Methods

All studies have been approved by Beijing Anzhen Hospital Ethics Committee and performed in accordance with the ethical standards.

### Animals

**Figure 1:**
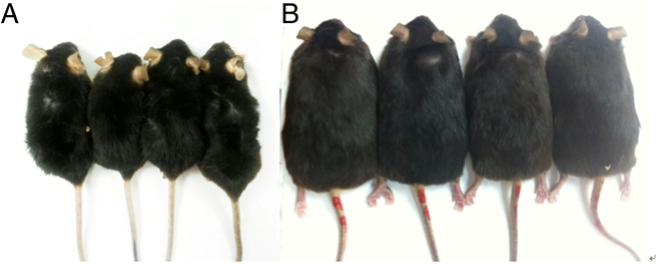
**12-wk male non-diabetic C57BL/6 mice (A) and age-matched diabetic db/db mice (B).**

Experiments were performed on 8-, 12- and 16-wk male homozygous diabetic (db/db) (n = 8 in each week) and age-matched heterozygote non-diabetic (Control) mice (n = 8 in each week), obtained from Beijing University (Beijing, China). The control and diabetic mice (Figure 1) used in this study were the C57BL/6J strain. The mice were housed in groups and allowed ad libitum access to water and standard laboratory mouse chow. All the experimental procedures described below adhered strictly to the guidelines set forth by the National Science and Technology Commission of China and approved by the institutional ethics committee. The experiment conformed to the Animal Research: Reporting In Vivo Experiments (ARRIVE) guidelines.

### Conventional echocardiographic measurements

Before examination, all mice were anesthetized in a closed chamber with 3% isoflurane in oxygen for 2 to 5 minutes until immobile. Each mouse was weighed, carefully shaved and taped supine to ECG electrodes on a heated procedure board with isoflurane (at 2%) supplied by a nose cone connected to the anesthesia machine. Warm ultrasound transmission gel was liberally applied to ensure optimal image quality. Echocardiography was performed using a 18 to 38 MHz linear-array transducer with a digital ultrasound system (Vevo2100 Imaging System, VisualSonics, Toronto, Canada). Standard two-dimensional gray-scale image and M-mode image at the mid-papillary level in the parasternal left ventricle short-axis view were obtained during each echocardiographic examination. Conventional echocardiographic image measurements were performed offline. All image acquisitions and offline measurements were conducted by a single investigator who was blinded to animal groups. All M-mode measurements were performed in end-diastole and end-systole according to the leading edge-to-leading edge method of the American Soiety of Echocardiography [21]. Conventional measurements of the left ventricle (LV) included: end-diastolic diameter (LVEDD), end-systolic diameter (LVESD), interventricular septal thickness (IVST) and posterior wall thicknesses (LVPWT), ejection fraction (LVEF), fractional shortening (LVFS).

LV mass =1.053 × [(LVEDD + LVPWT + IVST)^3^–LVEDD^3^]. All data are the average of at least two separate scans, each scan representing the average of three selected beats.

### Novel speckle tracking echocardiography (STE) based strain measures of myocardial deformation

**Figure 2:**
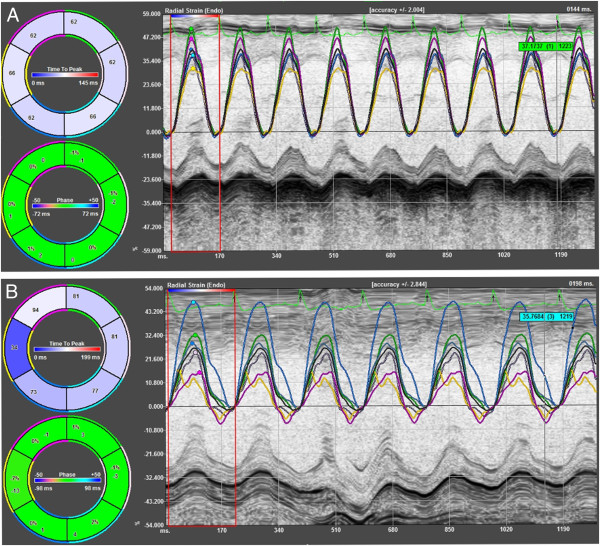
**Radial strain curves in 16-wk non-diabetic C57BL/6 mice (A) and age-matched diabetic db/db mice (B).**

**Figure 3:**
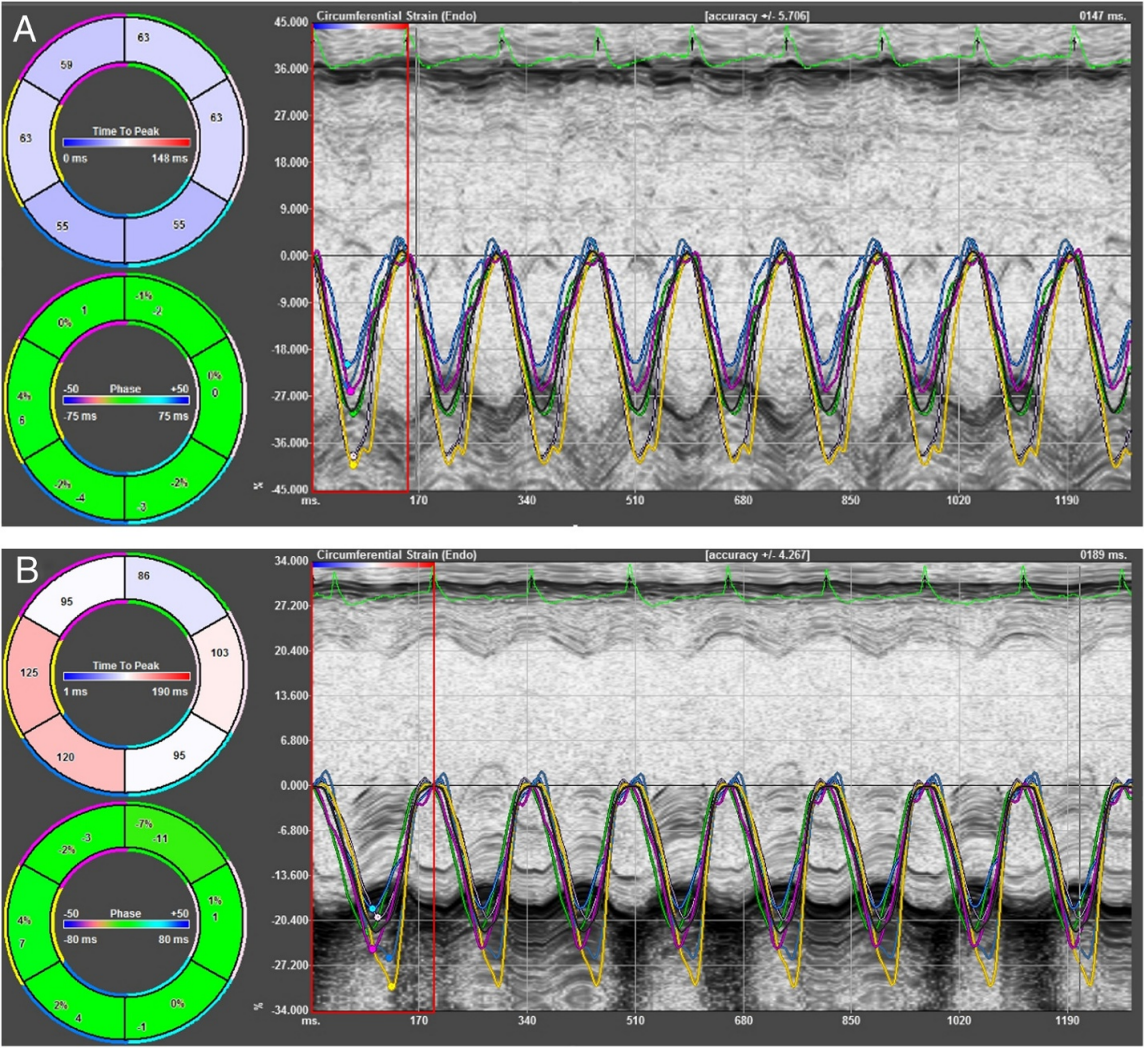
**Circumferential strain curves in 16-wk non-diabetic C57BL/6 mice (A) and age-matched diabetic db/db mice (B).**

Using STE based strain analysis of 2D gray scale echocardiographic images acquired from the parasternal short-axis views (at the mid-papillary level), strain analyses were quantified in the radial and circumferential axes. All images were acquired at a frame rate of >200 frames per second and at an average depth of 11 mm. Strain analyses were conducted by the same trained investigator on all animals according to the protocol detailed in the Online Data Supplement and using a speckle-tracking algorithm provided by VisualSonics (VevoStrain, VisualSonics). In brief, suitable B-mode loops were selected from digitally acquired echocardiographic images based on adequate visualization of the endocardial border and absence of image artifacts. Three consecutive cardiac cycles were selected for analysis based on image quality. Semiautomated tracing of the endocardial and epicardial borders were performed and verified over all 3 cardiac cycles and then corrected as needed to achieve good quality tracking throughout each cine loop. Tracked images were then processed in a frame-by-frame manner for strain measurements. Strain measures were averaged over the obtained cardiac cycles, resulting in curvilinear strain data (Figures 2 and 3). Short-axis view of the LV myocardium was divided into 6 standard anatomic segments for regional speckle-tracking based strain analysis throughout the cardiac cycle. Peak radial and circumferential strain measurements were recorded from each of the 6 standard segments in each view, providing regional strain values. For global radial and circumferential strain values, peak strain measurements were averaged across all 6 segments.

### Glucose, heart weight and body weight measurements

Mice were fasted for 8-hrs during the light cycle and blood was drawn from the tail vein. Blood glucose was measured using the glucometer (Johnson & Johnson).

After ultrasound detection, mice were sacrificed. Immediately after death, wet heart weight (HW) and body weight (BW) were measured.

### Statistical analysis

All analyses were performed with the use of SPSS 16.0 software. Resulting values are expressed as mean ± SD. Differences in conventional echocardiographic parameters and strain parameters between diabetic (db/db) and control mice of the same age were determined by means of an unpaired t test. Unpaired one-way ANOVA was used to test all parameters in different weeks of mice from the same genotype. Differences between means were regarded as statistically significant at *P* value <0.05.

## Results

### Body weight, heart weight, blood glucose and heart rate measurements

Body weight (BW), heart weight (HW), blood glucose and heart rate data of db/db and control mice (n = 8 for each group) were summarized in Table 1. The diabetic mice had larger body weight and fasting blood glucose compared to control mice at 8, 12 or 16 weeks of age. There were no difference in heart weight and heart rate between db/db mice and age-matched controls (P > 0.05). HW/BW of db/db mice significantly decreased compared to age-matched controls because of increased BW (P < 0.01).

**Table 1:** **Characteristics of control and db/db mice**

Variables	Control	Db/db	***P***value
**8 weeks**			
Body Weight (g)	22.26 ± 1.08	40.19 ± 1.96	*P < 0.0001*
Heart Weight (g)	0.12 ± 0.01	0.12 ± 0.01	*P > 0.05*
HW/BW	0.005 ± 0.0009	0.003 ± 0.0004	*P < 0.0001*
fasting blood glucose(mmol/L)	2.52 ± 0.50	7.62 ± 1.15	*P < 0.0001*
**12 weeks**			
Body Weight (g)	25.71 ± 1.41	47.82 ± 2.67	*P < 0.0001*
Heart Weight (g)	0.13 ± 0.01	0.13 ± 0.01	*P > 0.05*
HW/BW	0.005 ± 0.0002	0.003 ± 0.0002	*P < 0.0001*
fasting blood glucose(mmol/L)	2.15 ± 0.57	8.12 ± 1.22	*P < 0.0001*
**16 weeks**			
Body Weight (g)	27.14 ± 1.95	43.42 ± 2.05	*P < 0.0001*
Heart Weight (g)	0.13 ± 0.02	0.12 ± 0.01	*P > 0.05*
HW/BW	0.005 ± 0.0006	0.002 ± 0.0002	*P < 0.0001*
fasting blood glucose(mmol/L)	2.66 ± 0.29	12.71 ± 2.47	*P < 0.0001*

The table shows the diabetic mice have larger body weight and fasting blood glucose compared to control mice at 8, 12 or 16 weeks of age.

### Conventional echocardiographic measurements of left ventricle structure and systolic function

Conventional echocardiographic parameters of left ventricle structure and systolic function in db/db and control mice were summarized in Table 2. Echocardiography showed that the thickness of interventricular septum and posterior wall were similar at 8, 12 or 16 weeks of age between db/db and control mice (P > 0.05). There was no significant difference in LVEDD, LVESD, LVEF, LVFS and LV mass at the same weeks of age between db/db and control mice (P > 0.05).

**Table 2:** **M-mode parameters of the LV (parasternal short-axis view) in control and db/db mice**

Variables	Control	Db/db	***P***value
**8 weeks**			
Heart Rate(bmp)	397.50 ± 25.46	378.22 ± 37.25	*P > 0.05*
IVST(mm)	0.71 ± 0.05	0.66 ± 0.05	*P > 0.05*
LVPWD(mm)	0.69 ± 0.06	0.68 ± 0.05	*P > 0.05*
LVEDD(mm)	3.79 ± 0.23	3.68 ± 0.28	*P > 0.05*
LVESD(mm)	2.41 ± 0.27	2.26 ± 0.23	*P > 0.05*
LVEF(%)	66.25 ± 7.43	69.15 ± 7.25	*P > 0.05*
LVFS(%)	36.44 ± 5.13	38.39 ± 5.58	*P > 0.05*
LV mass(mg)	90.39 ± 11.15	83.32 ± 10.89	*P > 0.05*
**12 weeks**			
Heart Rate(bmp)	392.50 ± 44.99	388.77 ± 39.02	*P > 0.05*
IVST(mm)	0.68 ± 0.06	0.71 ± 0.05	*P > 0.05*
LVPWD(mm)	0.70 ± 0.06	0.69 ± 0.06	*P > 0.05*
LVEDD(mm)	3.90 ± 0.26	3.82 ± 0.18	*P > 0.05*
LVESD(mm)	2.49 ± 0.31	2.37 ± 0.20	*P > 0.05*
LVEF(%)	65.91 ± 7.54	68.51 ± 5.25	*P > 0.05*
LVFS(%)	36.01 ± 5.38	37.99 ± 4.53	*P > 0.05*
LV mass(mg)	93.21 ± 9.52	92.24 ± 13.44	*P > 0.05*
**16 weeks**			
Heart Rate(bmp)	381.25 ± 21.94	391.44 ± 39.81	*P > 0.05*
IVST(mm)	0.72 ± 0.03	0.71 ± 0.03	*P > 0.05*
LVPWD(mm)	0.69 ± 0.04	0.69 ± 0.05	*P > 0.05*
LVEDD(mm)	3.94 ± 0.19	3.77 ± 0.35	*P > 0.05*
LVESD(mm)	2.53 ± 0.21	2.36 ± 0.28	*P > 0.05*
LVEF(%)	66.78 ± 4.17	67.55 ± 7.79	*P > 0.05*
LVFS(%)	36.57 ± 3.30	37.25 ± 6.05	*P > 0.05*
LV mass(mg)	97.81 ± 8.31	90.98 ± 14.37	*P > 0.05*

The diagram shows no significant difference in the measurements of heart rate, left ventricular dimensions, thickness and function parameters by M-mode echocardiography in dbdb and control mice at 8,12 and 16 weeks of age (P > 0.05).

LVAWT, LVPWT, LVEDD, LVESD, LVEF and LVFS did not change with age for both dbdb and C57BL/6 mice (P > 0.05).

### STE measurements of myocardial deformation

**Figure 4:**
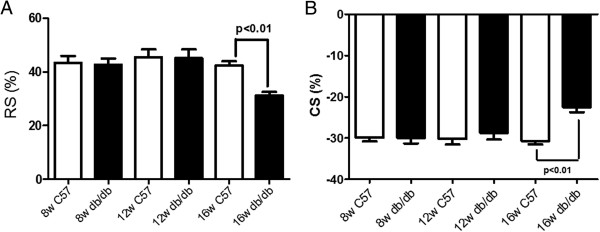
**The graphs shows at 8 and 12 weeks of age, there was no significant difference in left ventricular radial strain (A) and circumferential strain (B) between db/db mice and age-matched controls (P > 0.05).** At 16 weeks of age, radial strain **(A)** and circumferential strain **(B)** in db/db mice were lower than in control mice (P < 0.01). RS indicates radial strain; CS, circumferential strain; C57, non-diabetic C57BL/6 mice; db/db, db/db mice.

Using speckle tracking echocardiography, systolic global radial and circumferential strain values from the parasternal short-axis views at the mid-papillary level in db/db and control mice were obtained (Table 3 and Figure 4). At 8 and 12 weeks of age, there was no significant difference in left ventricular radial strain and circumferential strain between db/db mice and controls. But at 16 weeks of age, the left ventricular radial strain and circumferential strain in db/db mice were lower than in control mice (P < 0.01).

**Table 3:** **Radial and circumferential strain values in control and db/db mice by STE measurements**

Variables	Control	Db/db	***P***value
**8 weeks**			
Radial strain(%)	43.43 ± 7.03	42.73 ± 6.77	*P > 0.05*
Circumferential strain(%)	-29.92 ± 2.47	-29.95 ± 4.01	*P > 0.05*
**12 weeks**			
Radial strain(%)	45.43 ± 8.36	45.14 ± 9.75	*P > 0.05*
Circumferential strain(%)	-30.20 ± 3.83	-28.75 ± 4.82	*P > 0.05*
**16 weeks**			
Radial strain(%)	42.37 ± 4.40	31.19 ± 3.95	*P < 0.01*
Circumferential strain(%)	-30.71 ± 2.24	-22.54 ± 3.57	*P < 0.01*

The table shows at 8 and 12 weeks of age, there was no significant difference in left ventricular radial strain and circumferential strain between db/db mice and age-matched controls (P > 0.05). At 16 weeks of age, radial strain and circumferential strain in db/db mice were lower than in control mice (P < 0.01).

Left ventricular radial strain and circumferential strain did not change with age for C57BL/6J mice (P > 0.05). Comparison of 8- and 12-wk db/db mice, left ventricular radial strain and circumferential strain were significantly reduced in 16-wk db/db mice (P < 0.01).

## Discussion

Diabetic db/db mice provide one of more commonly used animal models used to investigate diabetic cardiomyopathy, with obesity and insulin resistance [16, 17]. In the literature, db/db mice develop severe dabetes by 8 weeks of age [22, 23]. Our study showed at 8, 12 and 16 weeks of age, diabetic (db/db) mice weighed significantly more than control mice. As expected, at 8 weeks of age diabetic mice had significantly elevated fasting blood glucose with hyperglycemia.

Diabetic cardiomyopathy is accompanied by functional, structural and pathologic cardiac characteristics. Echocardiography is a noninvasive method that can assess cardiac function in mice [24–26]. We used M-mode measurements of LV dimensions in end systole and end diastole and myocardium thickness to provide information on morphology and systolic function. However, we observed that left ventricular dimensions and myocardium thickness in db/db mice were not significantly increased until 16 weeks of age compared to control mice. The parameters reflecting the left ventricular systolic function such as LVEF and LVFS could not be detected unchanged in db/db mice. These findings were supported by Barouch and colleages as they reported echocardiographically in 24-week-old db/db mice on C57BL/6J background an unchanged LVEF and FS [27]. However, conventional echocardiographic measures lack sensitivity for capturing subtle variations in left ventricular (LV) performance. Changes in LV structure and global function, when detected by conventional echocardiographic parameters, are typically considered late manifestations of disease.

Speckle tracking echocardiography (STE) is a novel, non-Doppler-based technique used to detect myocardial wall motion and myocardial deformation. In clinical studies STE based on tissue deformation has provided an improved accuracy of myocardial contractility and quantification of global and regional myocardial function. Cardiovascular risk factors result in impaired strain measures before the development of decreased LVEF or ventricular dilatation [28, 29]. In experimental studies, the adoption of strain analysis in small animal models has been limited, primarily because of technical differences in imaging mice versus humans, including limited echocardiographic views, translational motion during image acquisition, and the effect of very high heart rates. However, recently developed speckle-tracking based techniques now allow for angle-independent, reproducible, and accurate strain measurements that may be applied to mice. STE can accurately predict pathophysiology during the evolution of MI [30, 31] and TAC-induced heart failure in mice [32]. LV contraction is a complex process involving deformation resulting in shortening in 3 normal directions; longitudinal, radial strain (RS) and circumferential strain (CS). RS and CS are more influenced by transmural fiber dysfunction (especially the midmyocardium), and are generally more suited for identifying dysfunction in ventricles with reduced left ventricle systolic function. Therefore our study used STE to assess systolic myocardial radial strain and circumferential strain with diabetic db/db and control mice. With the use of STE, we were able to detect lower myocardial radial and circumferential strain in db/db mice at 16 weeks of age, indicating LV contractile function was indeed abnormal in this type II diabetic mice. Our study showed that subtle abnormalities in strain were associated with the presence of diabetic cardiomyopathy even in the setting of normal cardiac structure and function by conventional measures. In other words, the present study therefore indicate that radial and circumferential strain are more sensitive and can be used for detection of early LV contractile dysfunction in this type II diabetic mice model.

It should be mentioned that the HRs play an important role in the determination of cardiac function in mice by echocardiography. Inhalation anesthesia with isoflurane has currently been considered ideal and useful for experimental studies in the mouse because of its rapid conduction, easy control of the depth of anesthesia, and stable HRs during observations [33, 34]. Usually, the range of HRs in anesthetic mice for echocardiography is 300 to 550 beats per minute (bpm), as reported [35]. In our experiment HRs were not significantly different between db/db mice and age-matched controls. Heart rate values of our mice were low but in this range. It may be related to anesthesia time.

## Conclusion

The present study shows that speckle tracking echocardiography can be used to evaluate cardiac functional alterations in mouse models of cardiovascular disease. Radial and circumferential strain are more sensitive and can be used for detection of early left ventricular contractile dysfunction in db/db type II diabetic mice.
